# Selective Design of Mesoporous Bi_2_Se_3_ Films with Orthorhombic and Rhombohedral Crystals

**DOI:** 10.1002/smll.202501534

**Published:** 2025-04-24

**Authors:** Minsu Han, Tomota Nagaura, Ho Ngoc Nam, Zihao Yang, Azhar Alowasheeir, Quan Manh Phung, Takeshi Yanai, Jeonghun Kim, Saad M. Alshehri, Tansir Ahamad, Yoshio Bando, Yusuke Yamauchi

**Affiliations:** ^1^ Department of Materials Process Engineering Graduate School of Engineering Nagoya University Furo‐cho, Chikusa‐ku Nagoya Aichi 464–8603 Japan; ^2^ Australian Institute for Bioengineering and Nanotechnology (AIBN) The University of Queensland Brisbane QLD 4072 Australia; ^3^ Department of Chemistry Graduate School of Science Nagoya University Furo‐cho, Chikusa‐ku Nagoya 464–8602 Japan; ^4^ Institute of Transformative Bio‐Molecules (WPI‐ITbM) Nagoya University Furo‐cho, Chikusa‐ku Nagoya 464–8602 Japan; ^5^ Department of Chemical and Biomolecular Engineering Yonsei University 50 Yonsei‐ro, Seodaemun‐gu Seoul 03722 South Korea; ^6^ Chemistry Department College of Science King Saud University P.O. Box 2455 Riyadh 11451 Saudi Arabia; ^7^ Australian Institute for Innovative Materials University of Wollongong Squires Way North Wollongong NSW 2500 Australia

**Keywords:** bismuth selenides, crystal phase, electrochemical glucose sensing, mesoporous materials, soft templating method

## Abstract

Materials with the same chemical composition can exhibit distinct properties depending on their crystal phases. Here, the synthesis of two types of mesoporous Bi_2_Se_3_ films at different reduction potentials is reported and their application in electrochemical glucose sensing. Mesoporous Bi_2_Se_3_ is synthesized by incorporating block copolymer micelle assemblies into the deposition solution and applying a reduction potential. To characterize the crystal phases accurately, Bi_2_Se_3_ films are heat‐treated at 200 °C for 1 h in a nitrogen atmosphere. The results reveal that the Bi_2_Se_3_ films synthesized under different conditions exhibit clearly distinct phases: rhombohedral (*R*‐Bi_2_Se_3_) and orthorhombic (*O*‐Bi_2_Se_3_). The *R*‐Bi_2_Se_3_‐8 nm, featuring 8 nm pores and synthesized at a more negative reduction potential, outperforms its nonporous counterpart, achieving a glucose sensing sensitivity of 0.143 µA cm^−2^ µM^−1^ and a detection limit of 6.2 µM at pH 7.4 in 0.1 M phosphate‐buffered saline solution. In contrast, the *O*‐Bi_2_Se_3_, prepared at a relatively positive potential, exhibits no glucose‐sensing activity. The inactivity of *O*‐Bi_2_Se_3_ for glucose oxidation is likely due to the energetically unfavorable intermediates, as predicted by density functional theory calculations. These findings underscore the critical role of crystal phase control in porous nanomaterials and pave the way for developing innovative porous systems.

## Graphical abstract

Two types of mesoporous Bi_2_Se_3_ films with distinct crystal phases—orthorhombic and rhombohedral—have been synthesized using a block copolymer‐based soft templating method. These mesoporous Bi_2_Se_3_ films exhibit different electrochemical responses toward glucose sensing. This study holds significant value as a pioneering attempt to control the lattice phase in mesoporous chalcogenides.

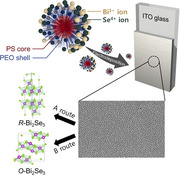

## 1 Introduction

Mesoporous materials, characterized by uniform pores ranging from 2 to 50 nm, have garnered significant attention for their exceptional performance in catalysts, sensors, and energy storage devices, owing to their large surface area and short diffusion paths.^[^ ^1^, ^2^, ^3^, ^4^, ^5^, ^6^, ^7^ ^]^ A widely used approach for synthesizing such materials is the block copolymer‐based soft templating method, which is a straightforward and efficient process.^[^ ^8^, ^9^, ^10^ ^]^ This method involves incorporating a polymer template into the reaction solution, reducing metal ions or precursors, and subsequently removing the polymer to create a porous structure.^[^ ^11^ ^]^ Using this technique, numerous mesoporous metal materials, such as Pt, Au, and Ag, with highly uniform pores, have been successfully synthesized, and their applications in catalytic active layers and optical sensors have been extensively demonstrated.^[^ ^12^, ^13^, ^14^ ^]^

Integrating mesoporous structures into chalcogenides, which are gaining increasing attention in the semiconductor and optical fields, is an area of active research.^[^ ^15^, ^16^ ^]^ Mesoporous chalcogenides are considered highly promising materials due to their large surface area and 3D pore networks, which enhance interactions with reactive species and dopants.^[^ ^17^, ^18^ ^]^ Additionally, their pore structures introduce novel optical properties, further expanding their potential applications.^[^ ^19^ ^]^ Notably, the use of block copolymer templates has been reported in several successful cases for synthesizing mesoporous chalcogenides such as CdSe, Cu_2_Se, and CuTe.^[^ ^20^, ^21^, ^22^ ^]^ This approach is particularly advantageous for its ability to achieve sophisticated pore architecture through a relatively simple and efficient process compared to other methods.

Although soft template‐based synthesis techniques have been proven effective for pore formation, few studies have explored the control of crystal phase or atomic arrangement in mesoporous materials. Given that the crystal phase in nanomaterials significantly influences band structure as well as chemical properties, precise control is essential for the development of novel materials. For instance, TiO_2_ can exist in three crystalline phases–anatase, rutile, and brookite–each exhibiting distinct photocatalytic performance.^[^ ^23^, ^24^, ^25^ ^]^ Similarly, the conductivity and optical properties of transition metal dichalcogenides depend on whether they adopt a hexagonal, trigonal, or distorted octahedral phase.^[^ ^26^, ^27^, ^28^ ^]^ By controlling the crystal phase in mesoporous materials, it is possible to achieve a synergistic effect, leveraging both the high surface area provided by the pore structure and the unique properties conferred by the crystal phase.

Bismuth selenide (Bi_2_Se_3_) has attracted interest as a semiconductor and n‐type thermoelectric material due to its favorable electronic properties and a large Seebeck coefficient.^[^ ^29^, ^30^, ^31^, ^32^ ^]^ Bi_2_Se_3_ crystallizes in two distinct phases: the thermodynamically stable rhombohedral phase and the metastable orthorhombic phase. The rhombohedral phase features a layered structure composed of quintuple layers stacked along the *c*‐axis, with a bandgap of ≈0.3 eV.^[^ ^33^, ^34^ ^]^ In contrast, the orthorhombic phase consists of distorted [BiSe_6_] octahedra arranged in a zigzag pattern and exhibits a larger bandgap of ≈1.2 eV.^[^ ^35^, ^36^, ^37^ ^]^ These two phases exemplify how materials with identical elemental compositions can display markedly different electronic and chemical properties. Due to the thermodynamic stability of the rhombohedral phase, most studies on Bi_2_Se_3_ have focused on this structure,^[^ ^38^, ^39^, ^40^ ^]^ while reports on the synthesis and applications of orthorhombic Bi_2_Se_3_ remain scarce. Recently, Luo et al. demonstrated a mechanism for synthesizing both rhombohedral and orthorhombic Bi_2_Se_3_ films via electrodeposition, providing a viable route for fabricating orthorhombic Bi_2_Se_3_ on various substrates under standard conditions.^[^ ^37^ ^]^

In our previous study, we successfully synthesized mesoporous Bi_2_Se_3_ films and reported their unique conductive properties.^[^ ^41^ ^]^ However, the crystal phase of the films was not clearly elucidated, limiting the interpretation of their chemical properties. In this study, we synthesize two types of mesoporous Bi_2_Se_3_ films–rhombohedral (*R*‐Bi_2_Se_3_) and orthorhombic (*O*‐Bi_2_Se_3_)–under different conditions and evaluate their performance in electrochemical glucose sensing. The small crystallite size of electrodeposited mesoporous Bi_2_Se_3_ makes precise identification of crystal phase challenging. To achieve a detailed analysis of lattice structure, the Bi_2_Se_3_ films are heat‐treated to enhance atomic ordering. This treatment results in distinct lattice phases, as observed in transmission electron microscopy (TEM) images and X‐ray diffraction (XRD) patterns, revealing that the two films consist of rhombohedral and orthorhombic phases, respectively. *R*‐Bi_2_Se_3_, synthesized at a negative reduction potential, exhibits electrochemical activity for glucose oxidation, whereas *O*‐Bi_2_Se_3_, synthesized at a relatively positive potential, shows no activity. Density functional theory (DFT) calculations suggest that the inactivity of *O*‐Bi_2_Se_3_ in glucose sensing arises from energetically unfavorable intermediates. The findings of this study underscore the critical role of crystal phase control in mesoporous chalcogenide materials and provide a strategic framework for tailoring crystal phases in mesoporous materials synthesized via the soft templating method.

## 2 Result and Discussion

### 2.1 Synthesis of Mesoporous Bi_2_Se_3_ using a Soft Templating Method

Mesoporous Bi_2_Se_3_ films are fabricated using a soft templating method based on block copolymer micelle assembly. Polystyrene‐block‐polyethylene oxide (PS‐b‐PEO), with a PS‐to‐PEO length ratio of 2:1 to 3:1, was fully dissolved in tetrahydrofuran (THF) to form a unimer solution. The polymer solution was then gradually introduced into the electrolyte, where it self‐assembled into micelles of uniform size. The homogeneous dispersion of polymer micelles is confirmed by the Tyndall effect (Figure S1, Supporting Information). In contrast, the rapid addition of the polymer solution leads to polymer aggregation and precipitation, preventing homogeneous dispersion.

**Figure 1:**
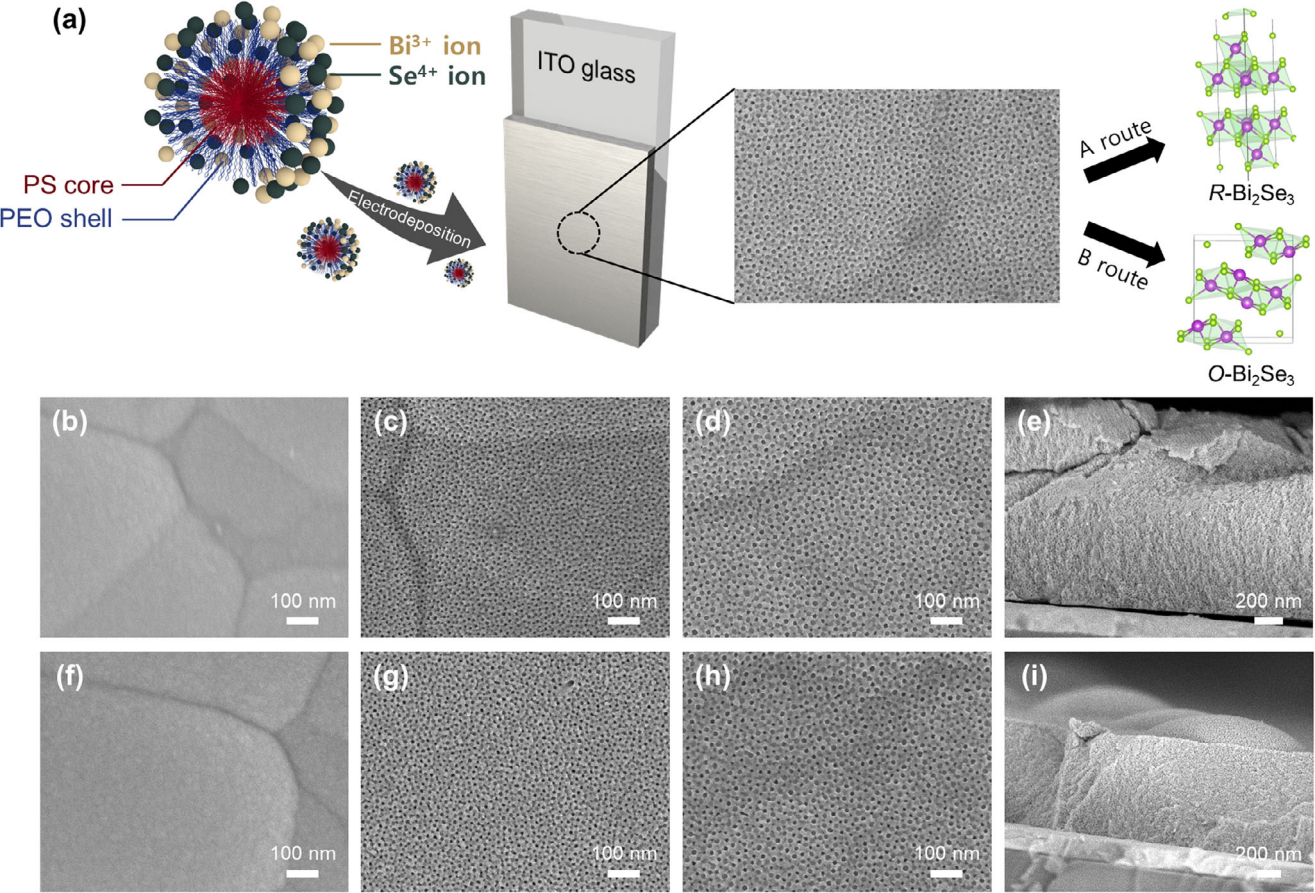
a) Schematic illustration of the electrodeposition of mesoporous Bi_2_Se_3_ films and the difference in crystal phase depending on the deposition conditions. b–d,f–h) Top‐view SEM images of (b–d) *R*‐Bi_2_Se_3_ films synthesized at −0.06 V and (f–h) *O*‐Bi_2_Se_3_ films synthesized at 0.00 V. (b,f) *R*‐Bi_2_Se_3_‐non and *O*‐Bi_2_Se_3_‐non prepared in the absence of polymer. c,g) *R*‐Bi_2_Se_3_‐8 nm and *O*‐Bi_2_Se_3_‐8 nm synthesized using PS_5000_‐b‐PEO_2500_. d,h) *R*‐Bi_2_Se_3_‐12 nm and *O*‐Bi_2_Se_3_‐12 nm synthesized using PS_9000_‐b‐PEO_3500_. e,i) Cross‐sectional SEM images of e) *R*‐Bi_2_Se_3_‐8 nm and i) *O*‐Bi_2_Se_3_‐8nm.

The micelles exhibit a core‐shell structure, with a hydrophobic PS core and a hydrophilic PEO shell. Precursor ions are thought to interact with the hydrophilic PEO chains (**Figure** **1**a). Upon applying a reduction potential, the precursor ions surrounding the micelles are reduced to form Bi_2_Se_3_. After electrodeposition, the polymer micelles were completely removed using THF, yielding a mesoporous Bi_2_Se_3_ film with uniformly sized pores distributed throughout the structure. Fourier transform infrared (FTIR) spectroscopy confirms that PS‐b‐PEO present in the mesoporous Bi_2_Se_3_ film before washing is completely removed after washing, as evidenced by the disappearance of its characteristic peaks (Figure S2, Supporting Information). Furthermore, aside from peaks attributed to CO_2_ adsorption (2339 and 2360 cm^−1^), no additional peaks are observed, indicating that no chemical species are adsorbed on the surface of the mesoporous Bi_2_Se_3_ film.

It has been reported that reduction potential, substrate type, and temperature influence the crystal structure of Bi_2_Se_3_ during electrodeposition.^[^ ^35^, ^42^, ^43^, ^44^ ^]^ Previous studies have demonstrated that the structure of the seed layer in the initial nucleation process plays a crucial role, ultimately determining the crystal phase of the synthesized Bi_2_Se_3_.^[^ ^37^, ^42^ ^]^ In this study, different reduction potentials were applied during seed formation and film growth to fabricate two types of mesoporous Bi_2_Se_3_ films.

To investigate the electrodeposition behavior of Bi_2_Se_3_, cyclic voltammetry (CV) was performed. All potentials mentioned in this manuscript are referenced against Ag/AgCl/saturated KCl. The electrochemical reaction was examined by applying a potential from −0.5  to 0.5 V to an indium tin oxide (ITO) glass substrate immersed in an electrolyte solution containing Bi(NO_3_)_3_, SeO_2_, and PS‐b‐PEO micelles (Figure S3, Supporting Information). In the first cycle, an onset potential of −0.06 V and a reduction peak at −0.12 V are observed, corresponding to the formation of a seed layer for Bi_2_Se_3_ synthesis. In the second cycle, the reduction peak shifts in the positive direction, with a new peak appearing at 0.00 V, indicating that the preformed seed layer facilitates Bi_2_Se_3_ growth. The CV curve of the electrolyte containing only Bi(NO_3_)_3_ without SeO_2_, exhibits a reduction peak at −0.30 V and an oxidation peak at 0.19 V in the first cycle, which shift to −0.16  and 0.17 V, respectively, in the second cycle (Figure S4a, Supporting Information). However, Se reduction occurs at potentials lower than −0.4 V (Figure S4b, Supporting Information). The shift in the Bi reduction peak (from the 1st cycle to the 2nd cycle) in Figure S4a (Supporting Information) follows a similar trend to that observed in the CV curve of the electrolyte containing both Bi(NO_3_)_3_ and SeO_2_ (Figure S3, Supporting Information), indicating that Bi plays a crucial role in the synthesis of Bi_2_Se_3_.

For rhombohedral *R*‐Bi_2_Se_3_, the seed layer was formed by sweeping the potential from the open‐circuit potential of 0.4  to −0.5 V and then to 0.5 V using CV, yielding a seed with a relatively low Bi ratio (Figure S5a–c, Supporting Information). In contrast, for orthorhombic *O*‐Bi_2_Se_3_, the seed layer was formed by applying a potential sweep from 0.4  to −0.1 V (Figure S5d–f, Supporting Information). The ITO glass coated with the seed layer was removed from the electroltyte, gently wiped with a wiper, and then re‐immersed in the electrolyte solution. Subsequently, *R*‐Bi_2_Se_3_ films were synthesized by applying a continuous reduction potential of −0.06 V, while *O*‐Bi_2_Se_3_ films were grown at 0.00 V (Figure S4c, Supporting Information). When the reduction potential is applied immediately after depositing the Bi_2_Se_3_ seed layer on ITO glass, film growth is concentrated around the seed sites, leading to uneven films with limited thickness (Figure S6a, Supporting Information). This suggests that Bi_2_Se_3_ exhibits a low affinity to ITO glass. However, after initial seed formation, gently wiping the substrate with a wiper to disperse aggregated seeds results in a smooth and well‐defined Bi_2_Se_3_ surface (Figure S6b, Supporting Information). Directly applying the reduction potential to the ITO glass without a separate seed formation step results in a morphology similar to that observed when the substrate is not wiped after CV (Figure S6c, Supporting Information).

Despite being synthesized under different electrochemical conditions, *R*‐Bi_2_Se_3_ and *O*‐Bi_2_Se_3_ exhibit nearly identical morphologies in scanning electron microscopic (SEM) images. When PS_5000_‐b‐PEO_2500_ (with molecular weights of 5000 and 2500 for the PS and PEO blocks, respectively) is used, both *R*‐Bi_2_Se_3_ and *O*‐Bi_2_Se_3_ have an average pore size of 7.7 nm (Figure 1c,g; Figure S7a, Supporting Information), and they are designated as *R*‐Bi_2_Se_3_‐8 nm and *O*‐Bi_2_Se_3_‐8 nm, respectively. When PS_9000_‐b‐PEO_3500_ (with molecular weights of 9000 and 3500 for the PS and PEO blocks, respectively) is used, *R*‐Bi_2_Se_3_ exhibits an average pore size of 11.7 nm, while *O*‐Bi_2_Se_3_ has an average pore size of 12.0 nm (Figure 1d,h; Figure S7b, Supporting Information), and they are designated as *R*‐Bi_2_Se_3_‐12 nm and *O*‐Bi_2_Se_3_‐12 nm, respectively. Films prepared using an electrolyte without polymers exhibit smooth surfaces (Figure 1b,f) and are designated as *R*‐Bi_2_Se_3_‐non and *O*‐Bi_2_Se_3_‐non, respectively. Cross‐sectional SEM images confirm that *R*‐Bi_2_Se_3_‐8 nm and *O*‐Bi_2_Se_3_‐8 nm have uniformly distributed pores throughout the films (Figure 1e,i).

The uniformity of the pore structure was further confirmed by measuring the pore‐to‐pore distance using small‐angle X‐ray scattering (SAXS) analysis (Figure S8, Supporting Information).^[^ ^45^, ^46^ ^]^ *R*‐Bi_2_Se_3_‐8 nm and *O*‐Bi_2_Se_3_‐8 nm exhibit strong SAXS peaks at 0.434 and 0.477 nm^−1^, corresponding to pore‐to‐pore distances of 14.4 and 13.2 nm, respectively. These values are in good agreement with those measured directly from SEM images–14.4 nm for *R*‐Bi_2_Se_3_‐8 nm and 13.7 nm for *O*‐Bi_2_Se_3_‐8 nm (Figure S7c, Supporting Information). Similarly, *R*‐Bi_2_Se_3_‐12 nm and *O*‐Bi_2_Se_3_‐12 nm, synthesized using larger molecular weight block copolymers, show peaks at 0.285 nm^−1^ and 0.320 nm^−1^, corresponding to pore‐to‐pore distances of 22.6  and 19.4 nm, respectively. These also closely match the values obtained from SEM images–21.7 nm for *R*‐Bi_2_Se_3_‐12 nm and 19.0 nm for *O*‐Bi_2_Se_3_‐12 nm (Figure S7d, Supporting Information). As the pores are not arranged in a highly ordered or periodic fashion, no higher‐order peaks are observed in the SAXS patterns. These results confirm that the mesoporous Bi_2_Se_3_ films synthesized via electrochemical depositions possess uniformly distributed pores of consistent size throughout the film.

*R*‐Bi_2_Se_3_ and *O*‐Bi_2_Se_3_ films exhibit differences in appearance to the naked eye. *R*‐Bi_2_Se_3_, prepared at more negative potentials, displays a grayish and relatively rough surface, whereas *O*‐Bi_2_Se_3_ has a metallic and smooth appearance (Figure S9, Supporting Information). The difference in lattice structure or surface roughness resulting from the synthesis potential appears to have a greater influence on film appearance than the type of polymer used. However, factors such as the gentle wiping process after seed formation and the reduced contrast in appearance with longer deposition times suggest that multiple variables contribute to the final film morphology, warranting further investigation.

Regardless of the polymer type or synthesis conditions, all Bi_2_Se_3_ films exhibit an elemental ratio of Bi to Se between 39:61 and 41:59, as determined by energy‐dispersive X‐ray analysis (EDX) (Figure S10a, Supporting Information). The *R*‐Bi_2_Se_3_ films show a slightly higher Bi content (by ≈ 1%) due to their more negative reduction potentials, though this variation falls within the measurement error range. The elemental composition of the bottom surface of the Bi_2_Se_3_ films was analyzed by detaching *R*‐Bi_2_Se_3_‐8 nm and *O*‐Bi_2_Se_3_‐8 nm from the ITO glass using epoxy resin (Figure S10b, Supporting Information). The Bi content at the bottom surface is found to be slightly higher than at the upper surface, but the difference is within ≈1%, indicating that the mesoporous Bi_2_Se_3_ films maintain a uniform elemental composition throughout their thickness. Apart from minor surface irregularities caused by the detachment process, the pore structure at the bottom surface closely resembles that of the upper surface (Figure S10c,d, Supporting Information), confirming the structural consistency and robustness of the polymer micelle‐based soft templating method.

### 2.2 Analysis of Crystal Phases in Mesoporous Bi_2_Se_3_ Fims

**Figure 2:**
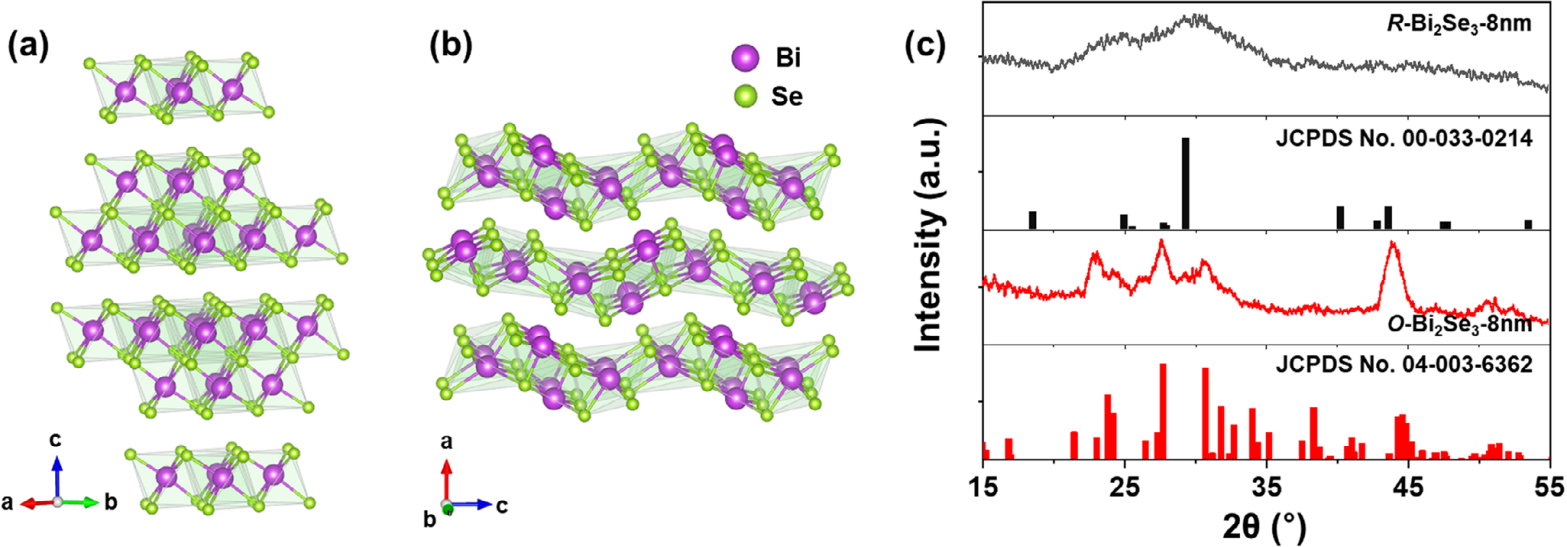
a,b) Schematic illustration of the atomic arrangement in (a) rhombohedral Bi_2_Se_3_ and (b) orthorhombic Bi_2_Se_3_. c) XRD patterns of *R*‐Bi_2_Se_3_‐8 nm and *O*‐Bi_2_Se_3_‐8 nm films deposited on ITO glass.

The crystal structures of the films were characterized by XRD. The *R*‐Bi_2_Se_3_‐8 nm film exhibits broad peaks, suggesting that it consists of small crystallites. The rapid electrochemical reduction and the presence of micelles may have hindered grain growth, leading to peak broadening. Although the broad peaks make definitive phase identification challenging, the *R*‐Bi_2_Se_3_‐8 nm film primarily adopts the rhombohedral phase (**Figure** **2**a,c). In contrast, the *O*‐Bi_2_Se_3_‐8 nm film displays relatively sharp peaks corresponding to the orthorhombic phase (Figure 2b,c). This may be due to its synthesis at relatively positive potential, which results in a slower reduction rate and facilitates the crystallization of the orthorhombic phase. A recent study also reported that electrodeposited orthorhombic Bi_2_Se_3_ exhibits sharper XRD peaks than its rhombohedral counterpart.^[^ ^37^ ^]^

**Figure 3:**
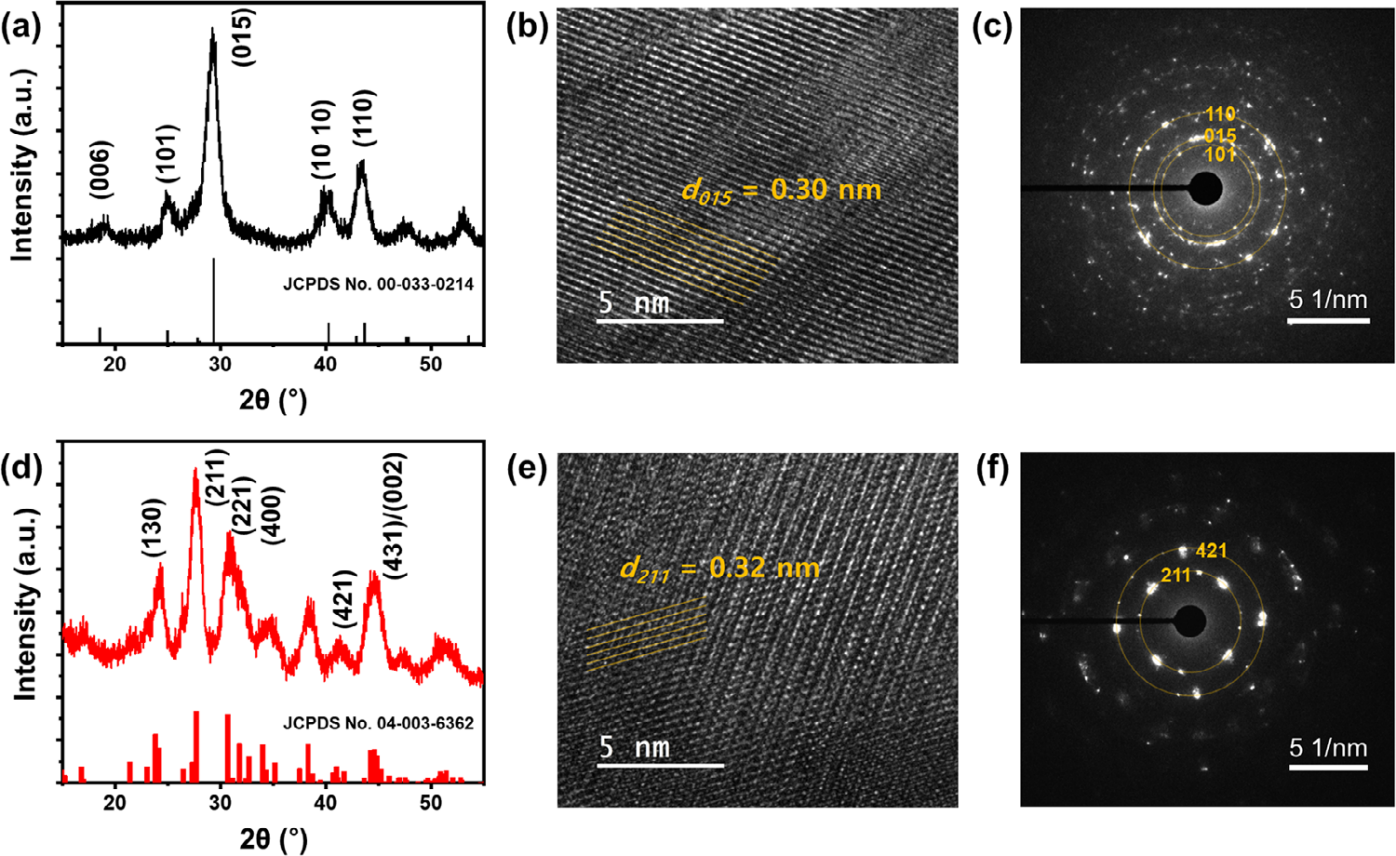
a,d) XRD patterns of (a) *R*‐Bi_2_Se_3_ and (d) *O*‐Bi_2_Se_3_ films annealed at 200 °C for 1 h under nitrogen gas, with a heating rate of 1 °C min^−1^. b,e) HRTEM images and c,f) corresponding SAED patterns of the annealed b,c) *R*‐Bi_2_Se_3_ and e,f) *O*‐Bi_2_Se_3_ films, respectively.

The XRD peak assignments above are mostly correct. However, to clearly identify the obtained crystal phases, thermal annealing was performed to promote atomic rearrangement in the films and to further analyze their lattice phases.^[^ ^47^ ^]^ When Bi_2_Se_3_ films are annealed at 200 °C for 1 h under nitrogen gas, the *R*‐Bi_2_Se_3_ film exhibits an intensified peak at 29.2°, confirming its rhombohedral phase (**Figure** **3**a). Similarly, the *O*‐Bi_2_Se_3_ film displays strong peaks corresponding to the orthorhombic phase after heat treatment (Figure 3d). The grain size of both films increases slightly while maintaining the trend observed in Figure 2c, suggesting that thermal annealing primarily facilitates atomic ordering without significantly altering phases. In the high‐resolution TEM (HRTEM) images, *R*‐Bi_2_Se_3_ exhibits lattice fringes with a spacing of 0.3 nm, corresponding to the (015) plane in rhombohedral Bi_2_Se_3_ (Figure 3b), which is consistent with the *d*‐spacing calculated from the XRD pattern. *O*‐Bi_2_Se_3_ displays lattice fringes with a spacing of 0.32 nm, corresponding to the (211) plane in orthorhombic Bi_2_Se_3_ (Figure 3e). Although both films exhibit polycrystalline characteristics, selected area electron diffraction (SAED) patterns indicate that *O*‐Bi_2_Se_3_ possesses higher crystallinity (Figure 3c,f).

To prevent structural collapse during annealing, the temperature was increased gradually at a rate of 1 °C min^−1^. As confirmed by SEM images (Figure S11, Supporting Information), this slow heating process preserves the pore structures in both films, allowing for enhanced crystallinity without significant atomic diffusion over long distances.

### 2.3 Electrochemical Glucose Sensing of Mesoporous Bi_2_Se_3_ Films

**Figure 4:**
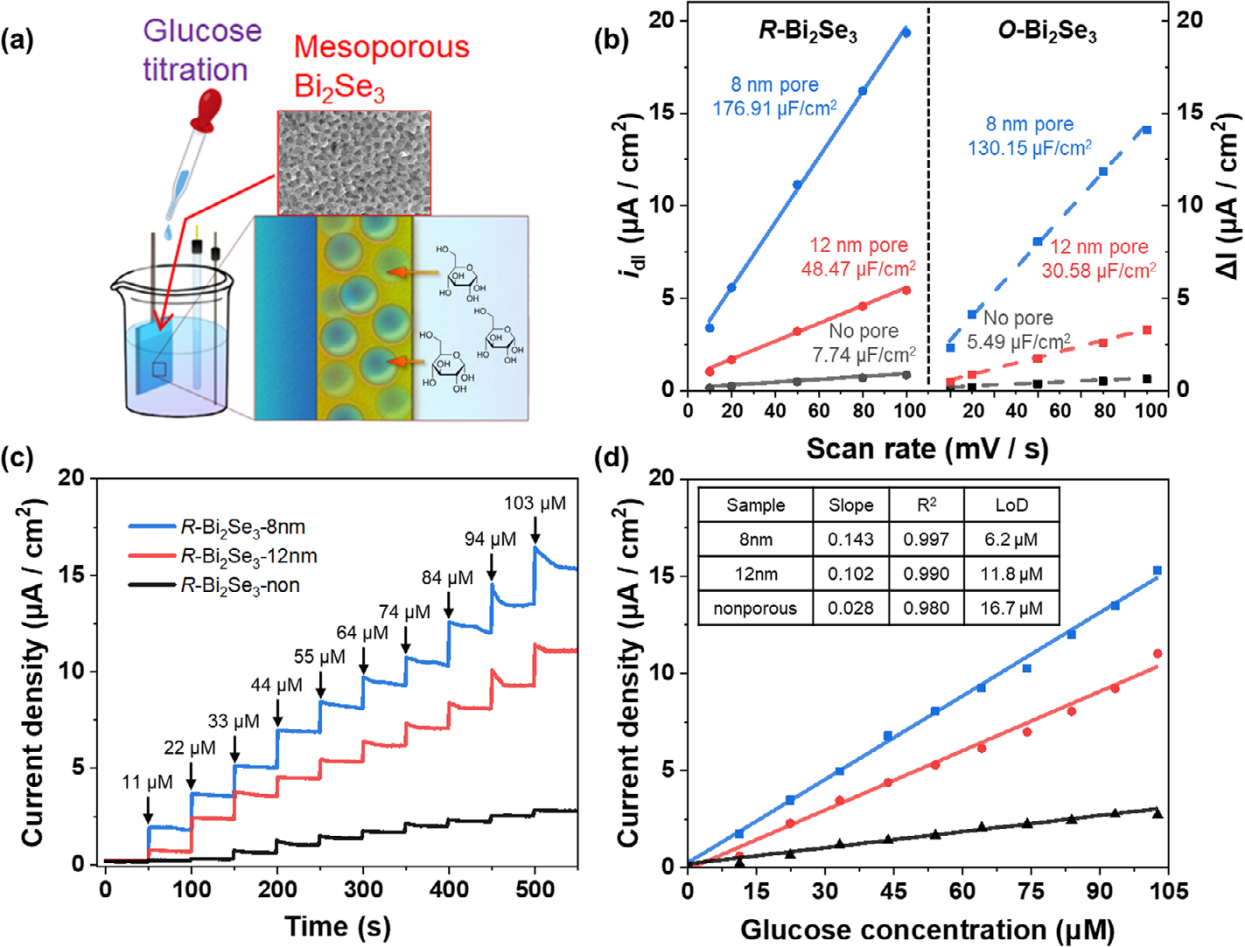
a) Schematic illustration of the electrochemical glucose detection experiment using mesoporous Bi_2_Se_3_ films. b) ECSA analysis of Bi_2_Se_3_ films with 8 nm pores, 12 nm pores, and nonporous structures in different crystal phases. c) Current response of *R*‐Bi_2_Se_3_ films during continuous titration of glucose solution in 0.1 M PBS (pH 7.4) at a constant potential of 0.1 V. d) Current density plot as a function of glucose concentration, demonstrating the sensing performance.

To evaluate the effect of pore structure and the applicability of the films for electrochemical applications, electrochemically active surface area (ECSA) measurements were conducted (**Figure** **4**b). Since the capacitance per unit area of Bi_2_Se_3_ is unknown, ECSA is indirectly assessed using the electrochemical double‐layer capacitance (*C*_dl_). *C*_dl_ was determined from CV cycles using 0.5 M H_2_SO_4_ solution as an electrolyte, in which the potential scan rate was varied from 10 to 100 mV s^−1^ in a non‐Faradaic region. In the *R*‐Bi_2_Se_3_ series, the *R*‐Bi_2_Se_3_‐8 nm and *R*‐Bi_2_Se_3_‐12 nm films exhibit 22.8‐fold and 6.26‐fold increases in ECSA, respectively, compared to the *R*‐Bi_2_Se_3_‐non. Similarly, in the *O*‐Bi_2_Se_3_ series, films with 8‐ and 12‐nm pores show 23.7‐fold and 5.57‐fold increases, respectively, compared to *O*‐Bi_2_Se_3_‐non. These results suggest that the presence of pores throughout the film enhances electrochemical activity by exposing more Bi_2_Se_3_ surface area.

To investigate the effects of surface area and crystal phase on electrochemical activity, Bi_2_Se_3_ films were applied to nonenzymatic glucose detection. Glucose levels are a critical indicator of human health, and their rapid and reliable detection is essential in both medical and daily‐life applications. Accordingly, glucose sensing is used here as a representative case to highlight the electrochemical activity of Bi_2_Se_3_. The glucose detection was performed in a 0.1 M phosphate‐buffered saline (PBS) solution at pH 7.4 to ensure practical applicability. The glucose detection response was analyzed using CV (Figure S12, Supporting Information). The *R*‐Bi_2_Se_3_ film exhibits an onset potential at −0.2 V and a reduction peak at 0.27 V, corresponding to glucose oxidation upon glucose addition. In contrast, the *O*‐Bi_2_Se_3_ film shows no new peaks despite glucose addition. The lack of glucose oxidation activity in O‐Bi_2_Se_3_ will be discussed later based on DFT calculations.

For glucose detection using *R*‐Bi_2_Se_3_, the detection potential was set at 0.1 V, and the current response to glucose concentration was analyzed through continuous titration of glucose solution (Figure 4a). All *R*‐Bi_2_Se_3_ films exhibit a sharp increase in current followed by a plateau upon glucose addition, as shown in Figure 4c. The decrease and subsequent plateau of the oxidation current, despite continuous stirring of the glucose solution, may be attributed to: 1) limited diffusion of glucose into the deep pores, 2) adsorption of gluconolactone, a product of glucose oxidation, onto the electrode surface, and 3) the depletion of glucose concentration near the Bi_2_Se_3_ surface as electrochemical oxidation proceeds. The film with 8‐nm pores displays the highest currents, outperforming films with larger or no pores. The current density of all samples increases linearly with glucose concentration (Figure 4d), with the 8‐nm pore sample exhibiting the steepest slope and high correlation coefficient, suggesting superior sensitivity and accuracy. The sensitivity of *R*‐Bi_2_Se_3_‐8 nm is determined to be 0.143 µA cm^−2^ µM^−1^, five times higher than that of the nonporous sample. The estimated limit of detection (LoD) for *R*‐Bi_2_Se_3_‐8 nm is 6.2 µM, three times lower than that of the nonporous sample (16.7 µM). The 12‐nm pore sample also exhibits improved sensitivity (0.102 µA cm^−2^ µM^−1^) and LoD (11.8 µM), primarily due to the increased surface area provided by the mesoporous structure. The lower‐than‐expected detection performance, despite the enhanced ECSA, may be attributed to diffusion limitations arising from the relatively large size of glucose molecules compared to hydrogen ions, as well as the reduction of active sites caused by residual impurities remaining after glucose oxidation.

### 2.4 DFT Analysis of the Glucose Oxidation Mechanism on Bi_2_Se_3_

DFT calculations were performed to elucidate the performance difference of mesoporous Bi_2_Se_3_ films in electrochemical glucose detection. Given the polymorphic nature of the material, four slab models are employed. Under normal conditions, Bi_2_Se_3_ adopts a rhombohedral structure; thus, three slab models are constructed from the rhombohedral Bi_2_Se_3_(0001) surface. The most stable surface, *R*(001)_Se1_, is relatively inert to oxidation due to the closed‐shell electronic structure of surface Se atoms,^[^ ^48^, ^49^, ^50^ ^]^ suggesting weak glucose adsorption. To explore alternative terminations, two additional slabs, *R*(001)_Bi_ and *R*(001)_Se2_, are generated by selectively cleaving the top Se and Bi layers of *R*(001)_Se1_, respectively. *R*(001)_Bi_ slab serves as a model for the Bi‐terminated layer that may form upon air exposure.^[^ ^51^, ^52^ ^]^ Additionally, we considered the metastable orthorhombic phase, selecting the (100) surface (labeled as *O*(100)) due to its lowest surface energy among low‐index surfaces.^[^ ^37^ ^]^

**Figure 5:**
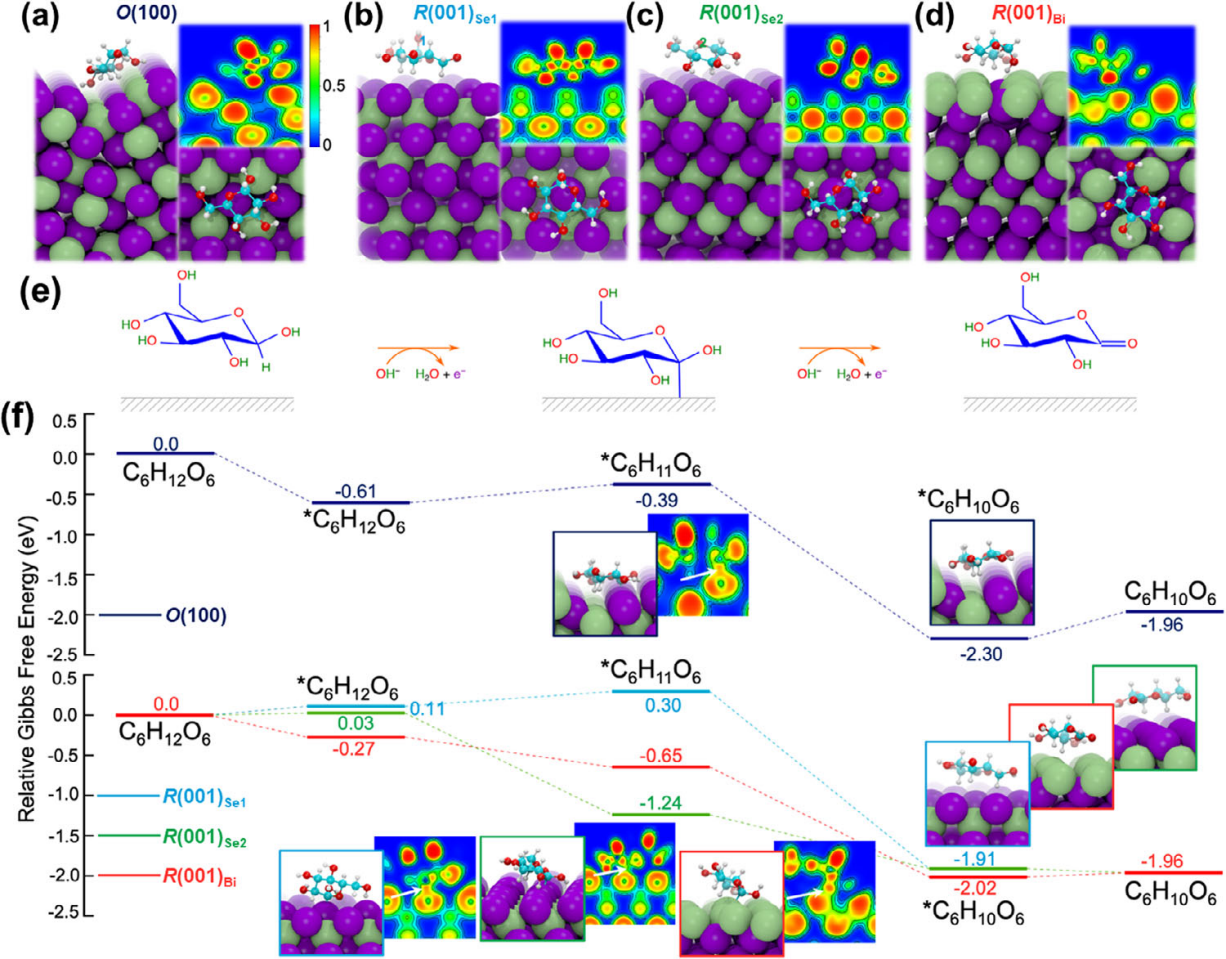
a–d) Optimized D‐glucose adsorption configurations shown from the side and top views, along with their corresponding ELF maps, on different Bi_2_Se_3_ surfaces: (a) *O*(100), (b) *R*(001)_Se1_, (c) *R*(001)_Se2_, and (d) *R*(001)_Bi_. Green, purple, cyan, red, and white spheres represent Bi, Se, C, O, and H atoms, respectively. e) Schematic representation of the glucose electrooxidation reaction to gluconolactone (C_6_H_10_O_6_). f) Calculated Gibbs free energy profiles for D‐glucose oxidation on *O*(100) (navy stepped line), *R*(001)_Se1_ (blue stepped line), *R*(001)_Se2_ (green stepped line), and *R*(001)_Bi_ (red stepped line) at 0.911 V versus reversible hydrogen electrode. The numbers (in eV) indicate the free energy levels relative to the initial state (^*^ + C_6_H_12_O_6_). Side views and ELF plots of the *C_6_H_11_O_6_ and *C_6_H_10_O_6_ intermediates are also shown. White arrows in the ELF plots highlight the covalent bond formation between *C_6_H_11_O_6_ and the surface.

The interaction between glucose and these surfaces was analyzed as it plays a critical role in electrochemical glucose detection. The binding energy (*E*_b_) of glucose was calculated as:

(1) $$E_{b}=E\left(*\mathrm{glu}\right)-E\left(\mathrm{glu}\right)-E\left(*\right)$$

where *E*(*glu) is the energy of glucose adsorbed on the surface, *E*(glu) is the energy of an isolated glucose molecule, and *E*(*) is the energy of the bare surface. The calculated binding energies for *R*(001)_Se1_, *R*(001)_Se2_, *R*(001)_Bi_, and *O*(100) are −0.56, −0.68, −0.97, and −1.29 eV, respectively. These binding energies, while significant, are primarily governed by van der Waals interactions. This is supported by the large separation between glucose and the surfaces, with the heavy atoms of glucose positioned at least 2.5 Å above the surface atoms (**Figure** **5**a–d). Furthermore, the very small electron localization function (ELF) values in the region between glucose and the surfaces confirm the predominance of van der Walls interactions.^[^ ^53^ ^]^ Among the three rhombohedral surfaces, *R*(001)_Se1_ is the most stable, with a surface energy of 12.5 meV Å^−2^, and consequently exhibits the weakest glucose binding. In contrast, *R*(001)_Se2_ and *R*(001)_Bi_ have significantly higher surface energies (67.0 and 72.2 meV Å^−2^, respectively), leading to stronger glucose adsorption. *O*(100) exhibits the strongest glucose binding (*E*_b_ = −1.29 eV), likely due to its zigzag structure, which aligns well with the chair confirmation of glucose. Given the dispersive nature of the glucose‐surface interaction, the high performance of our mesoporous materials is likely attributable to their large effective surface area and roughness. The increased surface area enhances glucose adsorption, thereby improving glucose detection sensitivity.^[^ ^54^, ^55^, ^56^ ^]^

Since glucose detection is closely linked to the material's ability to oxidize glucose, we investigated its oxidation into gluconolactone (C_6_H_10_O_6_) following the “activated chemisorption model” (Figure 5e).^[^ ^57^, ^58^, ^59^, ^60^, ^61^ ^]^ In this process, glucose adsorbs onto the surface and undergoes dehydrogenation at the hemiacetal carbon (C1), forming an intermediate (C_6_H_11_O_6_) bound to the surface. This intermediate is then further electrooxidized or oxidized by *OH in the double‐layer region, yielding adsorbed gluconolactone. The gluconolactone product subsequently desorbs and can undergo hydrolysis and neutralization to form gluconate.

In Figure 5f, we present the Gibbs free energy profile for the oxidation of D‐glucose:

(2) $$\begin{array}{c} {}*+C_{6}H_{12}O_{6}\to *C_{6}H_{12}O_{6}\to *C_{6}H_{11}O_{6}+\left(H^{+}+e^{-}\right)\to \\ {}*C_{6}H_{10}O_{6}+2\left(H^{+}+e^{-}\right)\to *+C_{6}H_{10}O_{6}+2\left(H^{+}+e^{-}\right) \end{array}$$

on all four investigated surfaces. The free energies of the species are referenced to * + C_6_H_12_O_6_. We first observe that free energy corrections consistently shift the adsorption energy of glucose by ≈0.7 eV, resulting in slightly positive adsorption free energy on the Se‐terminated surfaces. In contrast, glucose adsorption on *R*(001)_Bi_ and *O*(100) remains exergonic. On the two most stable surfaces, *R*(001)_Se1_ and *O*(100), glucose dehydrogenation is unfavorable, with Gibbs free energy values of ≈0.2 eV. Conversely, on the less stable surfaces *R*(001)_Se2_ and *R*(001)_Bi_, the dehydrogenation process is exergonic (Δ*G* = −1.21 and −0.38 eV, respectively), suggesting that the reaction should be favored.

In all cases, the adsorbed *C_6_H_11_O_6_ species forms a covalent bond with the surface via the C1 atom, with C1‐Se bond distances of 2.11, 2.36, and 2.15 Å on *R*(001)_Se1_, *R*(001)_Se2_, and *O*(100), respectively, and a C1‐Bi bond distance of 2.53 Å on *R*(001)_Bi_. The covalent nature of these bonds is evident from ELF analysis, which shows ELF values close to 1 in the region between C1 and the surfaces. The second dehydrogenation reaction, *C_6_H_12_O_6_ → *C_6_H_11_O_6_ + (H^+^ + e^−^), is exergonic on all surfaces. Except for the zigzag surface *O*(100), the final desorption step of gluconolactone (*C_6_H_10_O_6_) has a Gibbs free energy close to zero. Our analysis of all elementary reactions suggests that *R*(001)_Se1_ should exhibit the lowest electrooxidation activity for glucose, consistent with its lowest surface energy. This low activity is attributed to its weak ability to stabilize the dehydrogenated glucose radical. Among the four surfaces, *R*(001)_Se2_ and *R*(001)_Bi_ are expected to perform best: *R*(001)_Se2_ strongly stabilizes the intermediate *C_6_H_11_O_6_, while *R*(001)_Bi_ provides a balance by moderately binding both glucose and gluconolactone.

In conclusion, our theoretical investigation of different Bi_2_Se_3_ surfaces suggests that the high performance of mesoporous Bi_2_Se_3_ films for glucose detection arises from two key factors: (i) their high surface area, which enhances glucose physisorption, and (ii) the exposure of high‐energy reactive facets, which promote dehydrogenation reactions.

Based on DFT calculations, the lack of activity of *O*‐Bi_2_Se_3_ for glucose sensing can be attributed to the presence of energetically unfavorable intermediates. Specifically, two factors contribute to this limitation: (i) excessive stabilization of gluconolactone, which impedes its desorption and subsequent electrooxidation; and (ii) an energetically unfavorable glucose dehydrogenation step.

## 3 Conclusion

In this study, we have successfully synthesized mesoporous Bi_2_Se_3_ films with distinct crystal phases. Despite varying reduction conditions, Bi_2_Se_3_ films synthesized via the soft template method exhibit nearly identical pore structures. Films grown at more negative potentials on Bi‐poor seeds display a rhombohedral phase (*R*‐Bi_2_Se_3_), while those grown at more positive potentials on Bi‐rich seeds adopt an orthorhombic phase (*O*‐Bi_2_Se_3_). *R*‐Bi_2_Se_3_‐8 nm, characterized by numerous 8 nm‐sized pores, demonstrates a sensitivity of 0.143 µA cm^−2^ µM^−1^ and a detection limit of 6.2 µM in electrochemical glucose sensing using 0.1 M PBS solution as the electrolyte. These values are 5 and 3 times superior to those of the nonporous *R*‐Bi_2_Se_3_‐non sample (0.028 µA cm^−2^ µM^−1^ of sensitivity and 16.7 µM of detection limit), respectively. In contrast, *O*‐Bi_2_Se_3_ shows no activity in glucose sensing, attributable to its unfavorable energy state with the intermediate, as indicated by DFT calculations. This study is pioneering in its focus on crystal phase manipulation in mesoporous chalcogenide synthesis. It holds significant value due to the rarity of examples exploring the controlled synthesis of crystal phases and their electrochemical responses within materials of identical composition. We anticipate that this research will catalyze innovative approaches in nanomaterial design.

## 4 Experimental Section

### Chemicals

All chemicals and solvents were used as received without further purification. Bismuth(III) nitrate pentahydrate (Bi(NO_3_)_3_∙5H_2_O, ≥98%), selenium dioxide (SeO_2_, ≥99.9%), 70% nitric acid, D‐(+)‐glucose, and tetrahydrofuran (THF) were purchased from Sigma–Aldrich (USA). Block copolymers (BCPs) with different molecular weights, PS_9000_‐b‐PEO_3500_ and PS_5000_‐b‐PEO_2500_ (where the numbers indicate molecular weights), were obtained from Polymer Source Inc. All solutions were prepared with deionized water (DIW) treated with a Millipore water purification system.

### Characterization

The morphology and composition of the samples were analyzed using field emission scanning electron microscopy (FE‐SEM) equipped with energy‐dispersive X‐ray spectroscopy (EDS) (JSM‐7100F, JEOL Ltd.) at an accelerating voltage of 2.0 kV (15.0 kV for EDS). Pore sizes and pore‐to‐pore distances were measured from multiple SEM images of 8  and 12 nm samples using ImageJ software, with at least 100 measurements required for statistical accuracy. The microstructures were characterized by transmission electron microscopy (TEM) (JEM‐ARM200F “NEO ARM,” JEOL) at Yonsei Center for Research Facilities, Yonsei University, at an accelerating voltage of 200 kV. X‐ray diffraction (XRD) patterns were recorded using a SmartLab diffractometer (Rigaku Corp.) with Cu Kα radiation to determine the crystal structure. The lattice structure of Bi_2_Se_3_ was obtained from the Materials Project database (https://next‐gen.materialsproject.org/). Fourier transform infrared (FTIR) spectroscopy (FT/IR‐4X, JASCO, Japan) was performed in attenuated total reflection (ATR) mode over the range of 4000–400 cm^−1^ to confirm the removal of the polymer. Small‐angle X‐ray scattering (SAXS) measurements were performed in transmission mode using a Rigaku MicroMax‐007H to determine the pore‐to‐pore distances of the samples. Bi_2_Se_3_ film fragments, obtained by applying ultrasonic treatment to the electrodeposited films, were used for FTIR and SAXS analyses.

### Preparation of Electrolyte for Electrodeposition of Bi_2_Se_3_

Bi(NO_3_)_3_·5H_2_O was dissolved in 3 M nitric acid to obtain an 80 mM solution. SeO_2_ was dissolved in DIW at a concentration of 80 mM. A solution was prepared by sequentially mixing 0.6 mL of 80 mM Bi(NO_3_)_3_ solution with 3.1 mL of 3 M nitric acid and 0.7 mL of 80 mM SeO_2_ solution. PS_5000_‐b‐PEO_2500_ and PS_9000_‐b‐PEO_3500_ were completely dissolved in THF at a concentration of 40 mg mL^−1^. While stirring the mixture rapidly, 0.7 mL of THF was added, followed by the slow addition of 0.5 mL of the BCP solution. For the synthesis of *R*‐Bi_2_Se_3_‐8 nm and *O*‐Bi_2_Se_3_‐8 nm, PS_5000_‐b‐PEO_2500_ was used, while PS_9000_‐b‐PEO_3500_ was used for the synthesis of *R*‐Bi_2_Se_3_‐12 nm and *O*‐Bi_2_Se_3_‐12nm.

### Electrochemical Synthesis of Bi_2_Se_3_ Films

Electrochemical experiments, including cyclic voltammetry (CV) and electrodeposition, were performed using a three‐electrode setup with a CHI 760E electrochemical analyzer (CHI Instruments, USA). Indium tin oxide (ITO)‐coated glass, a platinum (Pt) wire, and an Ag/AgCl/saturated KCl electrode (RE‐1CP) served as the working, counter, and reference electrodes, respectively. ITO glass with a surface resistivity of 8–12 Ω sq^−1^ was purchased from Sigma–Aldrich, while the Pt wire and RE‐1CP were obtained from BAS Inc. (Japan). Before use, the ITO glass was cleaned via plasma treatment (Filgen, Japan).

For *R*‐Bi_2_Se_3_, a seed layer was formed by applying a potential from 0.4  to −0.5 V, followed by a further sweep to 0.5 V using CV. For *O*‐Bi_2_Se_3_, a seed layer was deposited by applying a potential from 0.4  to −0.1 V using linear sweep voltammetry. After seed layer formation, the working electrode surface was gently wiped with a wiper to disperse the seed. Films were then grown by applying −0.06 V for *R*‐Bi_2_Se_3_ and 0.00 V for *O*‐Bi_2_Se_3_ for 500 s. Following electrodeposition, the films were rinsed with 40 °C water to remove residual ions and subsequently immersed in 40 °C THF to dissolve the polymers. The washed films were then dried in a vacuum oven for 4 h.

To precisely identify the crystal phases of the films, the electrodeposited Bi_2_Se_3_ films were subjected to heat treatment in an electric furnace (AS ONE, Japan). Before heating, nitrogen gas was introduced at a flow rate of 200 cc min^−1^ for 10 min to establish an inert atmosphere inside the tube. The nitrogen flow rate was then reduced to 100 cc min^−1^, and the temperature was gradually increased to 200 °C at a rate of 1 °C min^−1^, followed by heat treatment at 200 °C for 1 h.

### ECSA Measurement and Electrochemical Glucose Sensing

The electrochemically active surface area (ECSA) was measured using a three‐electrode system consisting of a Bi_2_Se_3_‐coated ITO glass working electrode, a Pt wire counter electrode, and an Ag/AgCl reference electrode, with 0.5 M H_2_SO_4_ aqueous solution as the electrolyte. The ECSA was determined based on the electric double‐layer capacitance (*C*_dl_) of each sample. First, cyclic voltammetry (CV) was performed to identify the non‐Faradaic region where no redox reactions occur. The CV curve was then recorded by applying a potential of ±50 mV within each sample's optimal potential range. The potential sweep rate was varied from 10  to 100 mV s^−1^. A linear relationship was observed when the electric double‐layer current density (*i*_dl_) was plotted against the sweep rate (*v*), where *i*_dl_ was calculated as the average of the absolute values of the two current densities at the center of the potential window. The capacitance *C*_dl_ was determined using the following equation:

(3) $$C_{\mathrm{dl}}=d\left[i_{\mathrm{dl}}\right]/d\left[v\right]$$

Electrochemical glucose detection was conducted using 0.1 M phosphate‐buffered saline (PBS, pH 7.4) as the electrolyte. The oxidation potential of glucose on the Bi_2_Se_3_ film was determined by CV measurements in the potential range of −1.0 –0.6 V. For dynamic glucose detection, the current response was monitored while titrating glucose solution dropwise under a constant applied potential of 0.1 V.

### Computational Details

All periodic DFT calculations with the PBE‐GGA functional^[^ ^62^ ^]^ were performed with the Vienna Ab initio Simulation Package (VASP),^[^ ^63^, ^64^ ^]^ where the projector‐augmented wave (PAW) method^[^ ^65^, ^66^ ^]^ was employed with a kinetic energy cutoff of 450 eV. In addition, Grimme's dispersion correction with the Becke‐Johnson damping function (D3‐BJ) was included to account for weak van der Waals forces between the surfaces and adsorbates.^[^ ^67^ ^]^ Orthorhombic and rhombohedral Bi_2_Se_3_ unit cells were first fully optimized, giving lattice constants of *a* = *b* = 4.141 Å, *c* = 28.549 Å for rhombohedral Bi_2_Se_3_; and *a* = 11.666 Å, *b* = 4.112 Å, *c* = 11.442 Å for orthorhombic Bi_2_Se_3_. The lattice constants are in good agreement with experimental and previous DFT data.^[^ ^68^, ^69^ ^]^ All slab models were then constructed from the 3×3 and 3×1 unit cells of orthorhombic and rhombohedral structures, respectively. A vacuum region of 20 Å was then added along the *z*‐direction of slabs to eliminate the effect of self‐images owing to periodic boundary conditions. All slabs are stoichiometric, consisting of 36 Bi and 54 Se atoms. For the *R*(001) surfaces, 18 Bi and 27 Se bottom atoms were fixed to mimic the bulk nature. For the *O*(100), 12 Bi and 18 Se bottom atoms were fixed. The remaining atoms were allowed to fully relax until the residual forces were less than 0.03 eV Å^−1^. The irreducible Brillouin zone was sampled with Γ‐centered k‐meshes of 4×4×1.

The Gibbs free energy of each species was defined by *G* = *E* + *ZPE* ‐ *TS*, where *E* is the total DFT energy of a system, *ZPE* is the zero‐point energy correction, and *S* is the vibrational entropy of adsorbed intermediate. The free energy corrections were calculated at *p* = 1 atm and *T* = 298 K, using the VASPKIT package.^[^ ^70^ ^]^ For electrochemical steps involving a proton‐electron transfer, the computational hydrogen electrode model proposed by Nørskov et al. was applied, where *G* of (H^+^ + e^–^) is equal to *G* of 1/2H_2_(g).^[^ ^71^, ^72^, ^73^ ^]^ The effect of the applied potential (*U* = 0.911 V vs RHE, which corresponds to 0.3 V vs Ag/AgCl Sat. at pH 7) was approximated by adding Δ*G_U_* = ‒*neU* to the Gibbs free energy, where *n* is the number of electrons transferred and *e* is the elementary charge of an electron. For the adsorption of the intermediates, different adsorption configurations was studied but we reported only the most stable configurations.

## Conflict of Interest

The authors declare no conflict of interest.

## Supporting information



Supporting Information

## Data Availability

The data that support the findings of this study are available from the corresponding author upon reasonable request.
