# Supplementary material for: Selective Design of Mesoporous Bi2Se3 Films with Orthorhombic and Rhombohedral Crystals
Source: Small. 2025 Apr 24;21(23):2501534. doi: 10.1002/smll.202501534 (PMC12160694; doi:10.1002/smll.202501534)
Supplement: Supplementary file 1 — Supporting Information [file SMLL-21-2501534-s001.docx]

**Selective Design of Mesoporous Bi_2_Se_3_ Films with Orthorhombic and Rhombohedral Crystals**

Minsu Han, Tomota Nagaura, Ho Ngoc Nam, Zihao Yang, Azhar Alowasheeir, Quan Manh Phung, Takeshi Yanai, Jeonghun Kim, Saad M. A lshehri, Tansir Ahamad, Yoshio Bando, Yusuke Yamauchi*

Minsu Han, Ho Ngoc Nam, Zihao Yang, Azhar Alowasheeir, Yusuke Yamauchi

Department of Materials Process Engineering, Graduate School of Engineering, Nagoya University, Furo-cho, Chikusa-ku, Nagoya, Aichi 464-8603, Japan

Minsu Han, Tomota Nagaura, Yusuke Yamauchi

Australian Institute for Bioengineering and Nanotechnology (AIBN), The University of Queensland, Brisbane, Queensland 4072, Australia

E-mail: y.yamauchi@uq.edu.au

Quan Manh Phung, Takeshi Yanai

Department of Chemistry, Graduate School of Science, Nagoya University, Furo-cho, Chikusa-ku, Nagoya 464-8602, Japan

Quan Manh Phung, Takeshi Yanai

Institute of Transformative Bio-Molecules (WPI-ITbM), Nagoya University, Furo-cho, Chikusa-ku, Nagoya 464-8602, Japan

Jeonghun Kim, Yusuke Yamauchi

Department of Chemical and Biomolecular Engineering, Yonsei University, 50 Yonsei-ro, Seodaemun-gu, Seoul, 03722 South Korea

Saad M. A lshehri, Tansir Ahamad, Yoshio Bando

Chemistry Department, College of Science, King Saud University, P.O. Box 2455, Riyadh 11451, Saudi Arabia

Yoshio Bando

Australian Institute for Innovative Materials, University of Wollongong, Squires Way, North Wollongong, NSW 2500, Australia


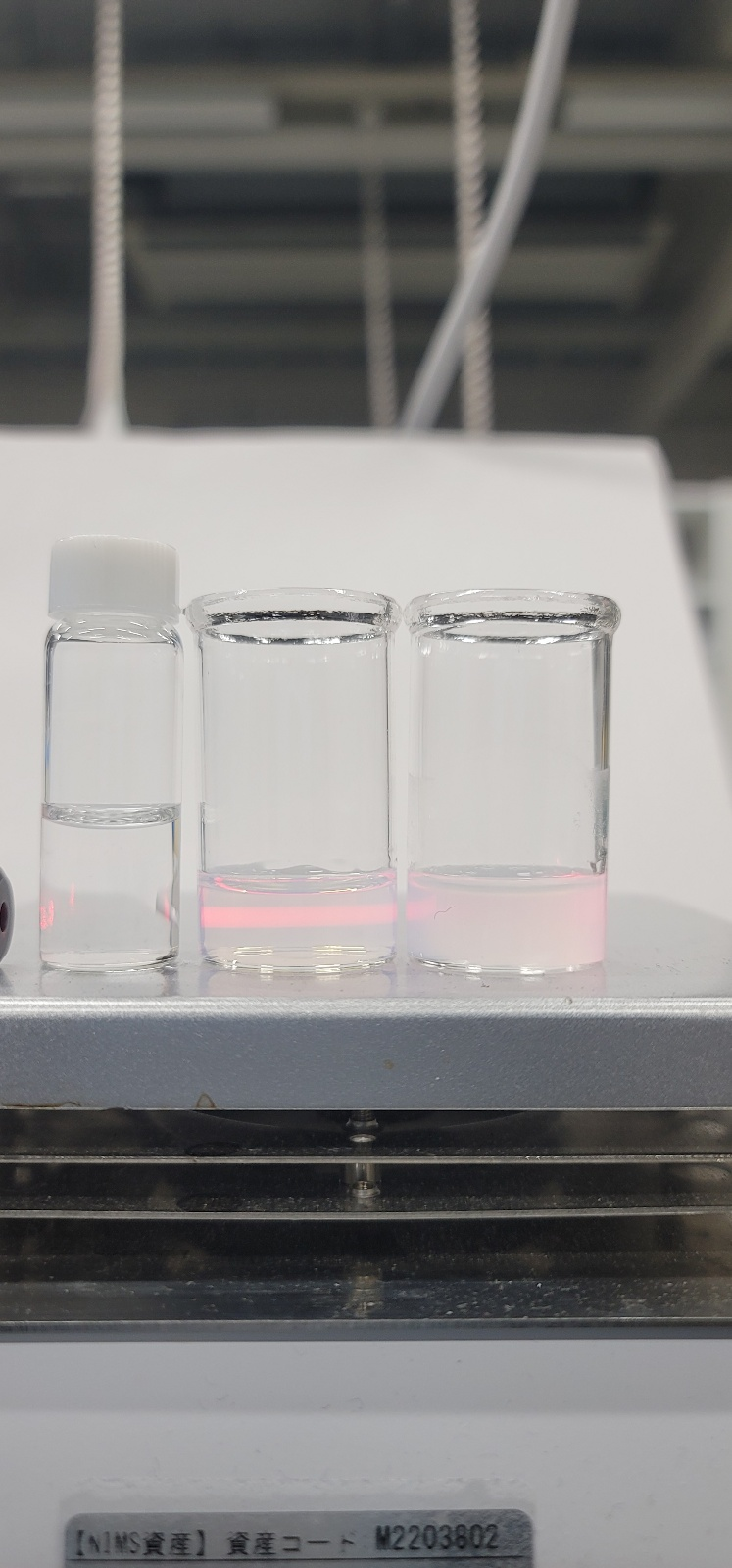


**Figure S1**. Tyndall effect observed in the electrolyte solution before polymer addition (left), after micelle formation upon polymer addition (middle), and in a solution where micelle formation was hindered due to polymer aggregation (right).


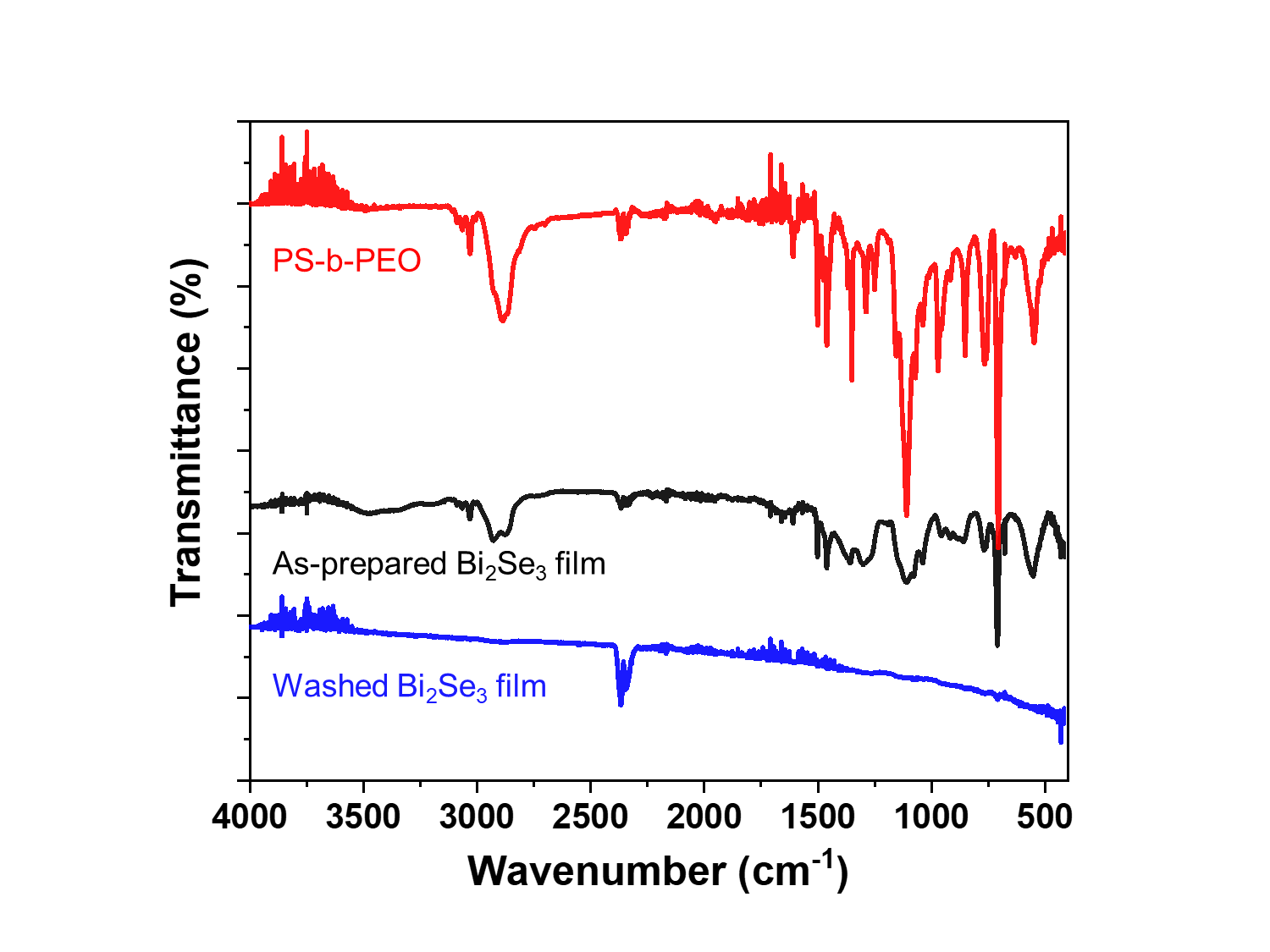


**Figure S2**. FTIR spectra of as-prepared mesoporous Bi_2_Se_3_ films (black), PS_5,000_-b-PEO_2,500_ (red), and washed Bi_2_Se_3_ films (blue), measured in ATR mode.





**Figure S3**. Cyclic voltammetry (CV) curves recorded in a potential window of −0.5 V to 0.5 V in a Bi_2_Se_3_ synthesis solution, using ITO glass, Pt wire, and Ag/AgCl electrode as the working, counter, and reference electrodes, respectively. The scan rate was 20 mV s^−1^.


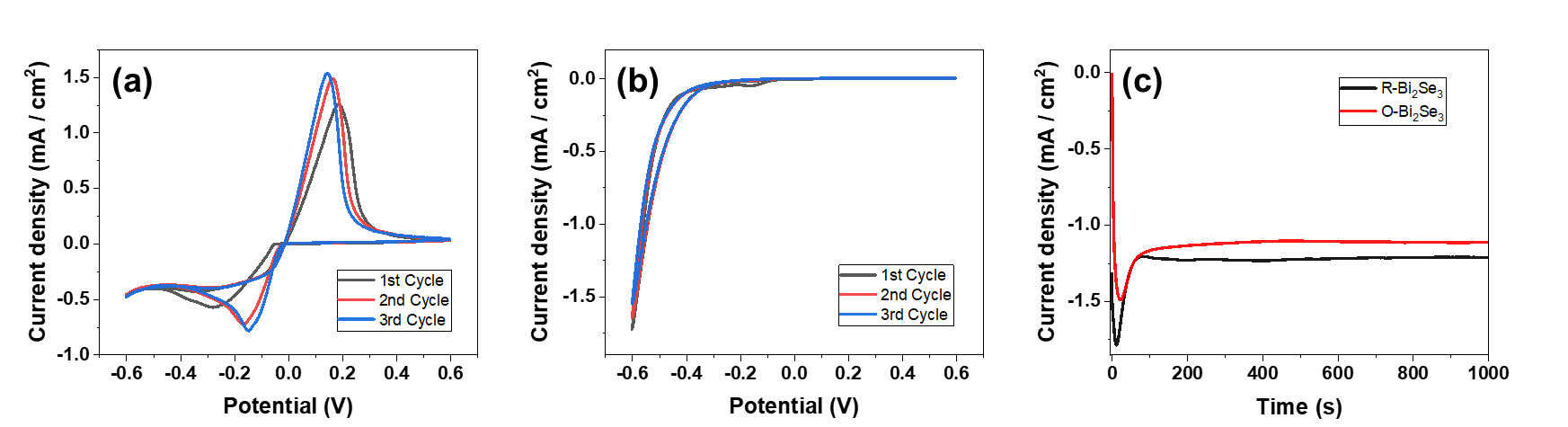
**Figure S4**. (a,b) CV curves of (a) an electrolyte containing Bi(NO_3_)_3_ and PS_5,000_-b-PEO_2,500_ and (b) an electrolyte containing SeO_2_ and PS_5,000_-b-PEO_2,500_. The scan rate was 20 mV s^−1^. (c) Amperometric *i*-*t* curves recorded during the synthesis of *R*-Bi_2_Se_3_ at −0.06 V and *O*-Bi_2_Se_3_ at 0.00 V.

**
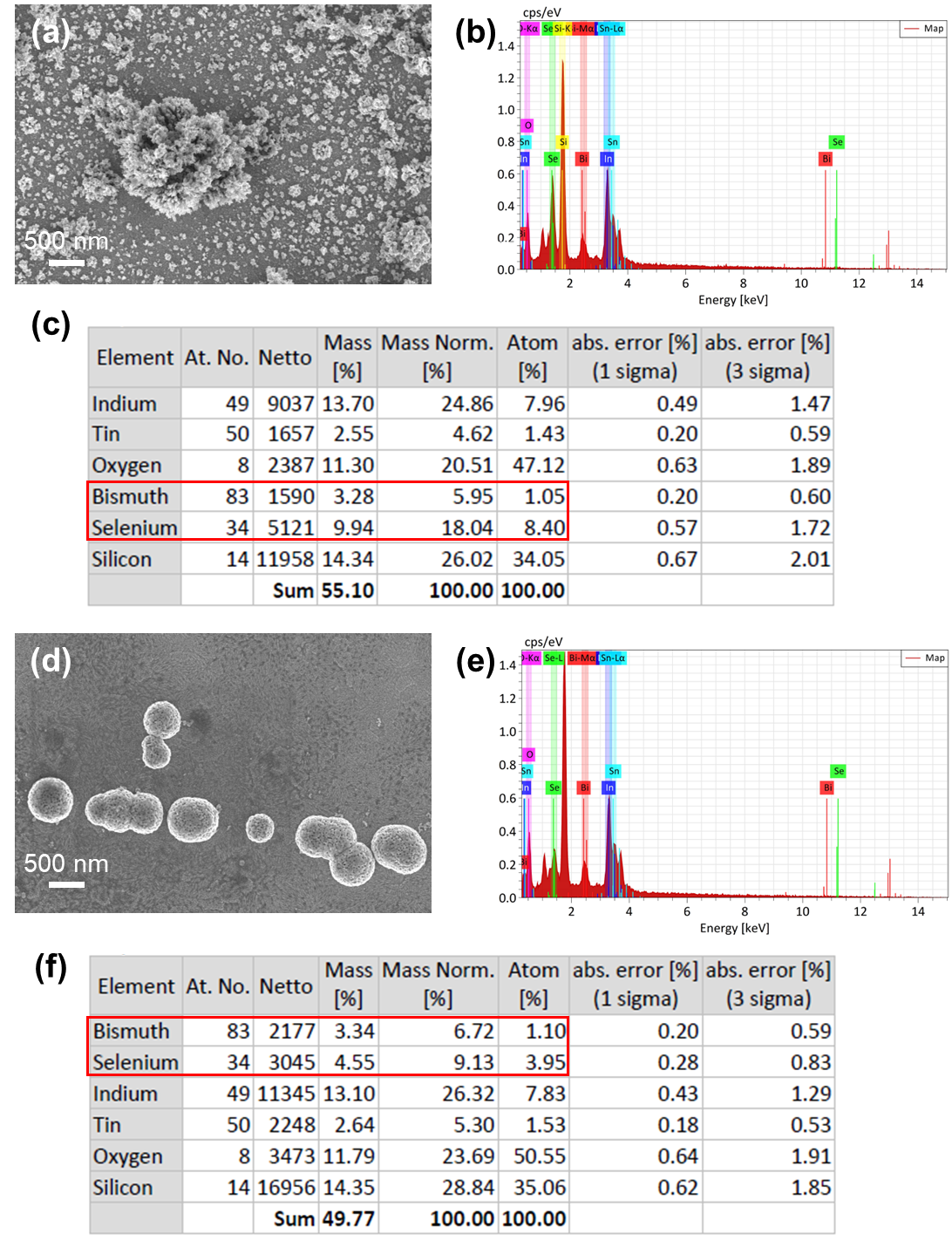
**

**Figure S5**. (a,d) Scanning electron microscopy (SEM) images and (b,c,e,f) energy dispersive X-ray analysis (EDX) of (a-c) the seed layer of *R*-Bi_2_Se_3_ formed by cyclic voltammetry (CV) from 0.4 to −0.5 V and then to 0.5 V and (d-f) the seed layer of *O*-Bi_2_Se_3_ formed by linear sweep voltammetry (LSV) from 0.4 V to −0.1 V.


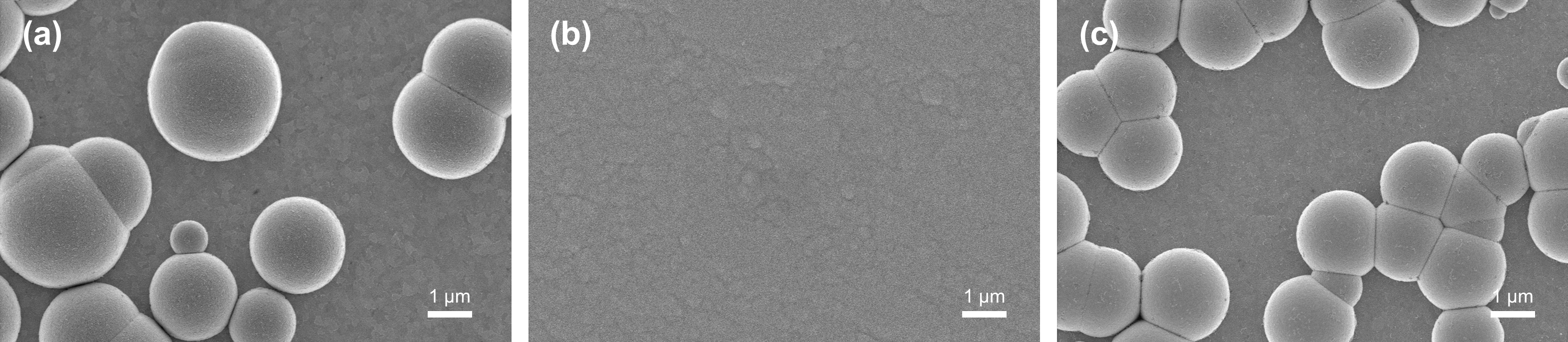
**Figure S6**. SEM images of (a) *R*-Bi_2_Se_3_ film synthesized by applying −0.06 V for 500 s immediately after seed formation *via* CV, (b) *R*-Bi_2_Se_3_ film synthesized after gently wiping the surface once following seed formation before applying the potential, and (c) *R*-Bi_2_Se_3_ film synthesized by directly applying −0.06 V for 500 s without a seed formation step.


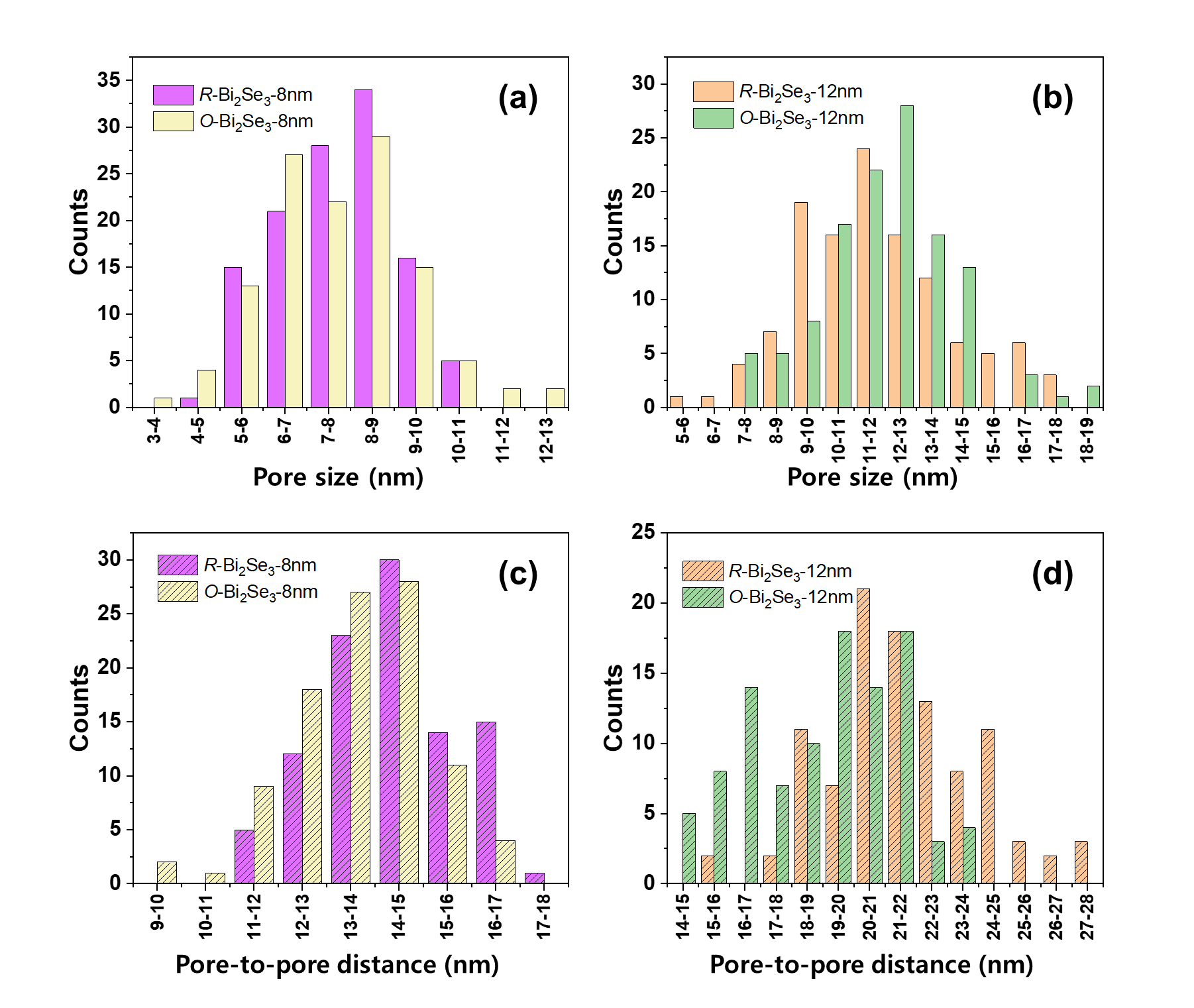


**Figure S7**. (a,b) Pore size distribution and (c,d) pore-to-pore distance distribution of mesoporous Bi_2_Se_3_ films prepared using (a,c) PS_5,000_-b-PEO_2,500_ and (b,d) PS_9,000_-b-PEO_3,500_, as determined from SEM images.


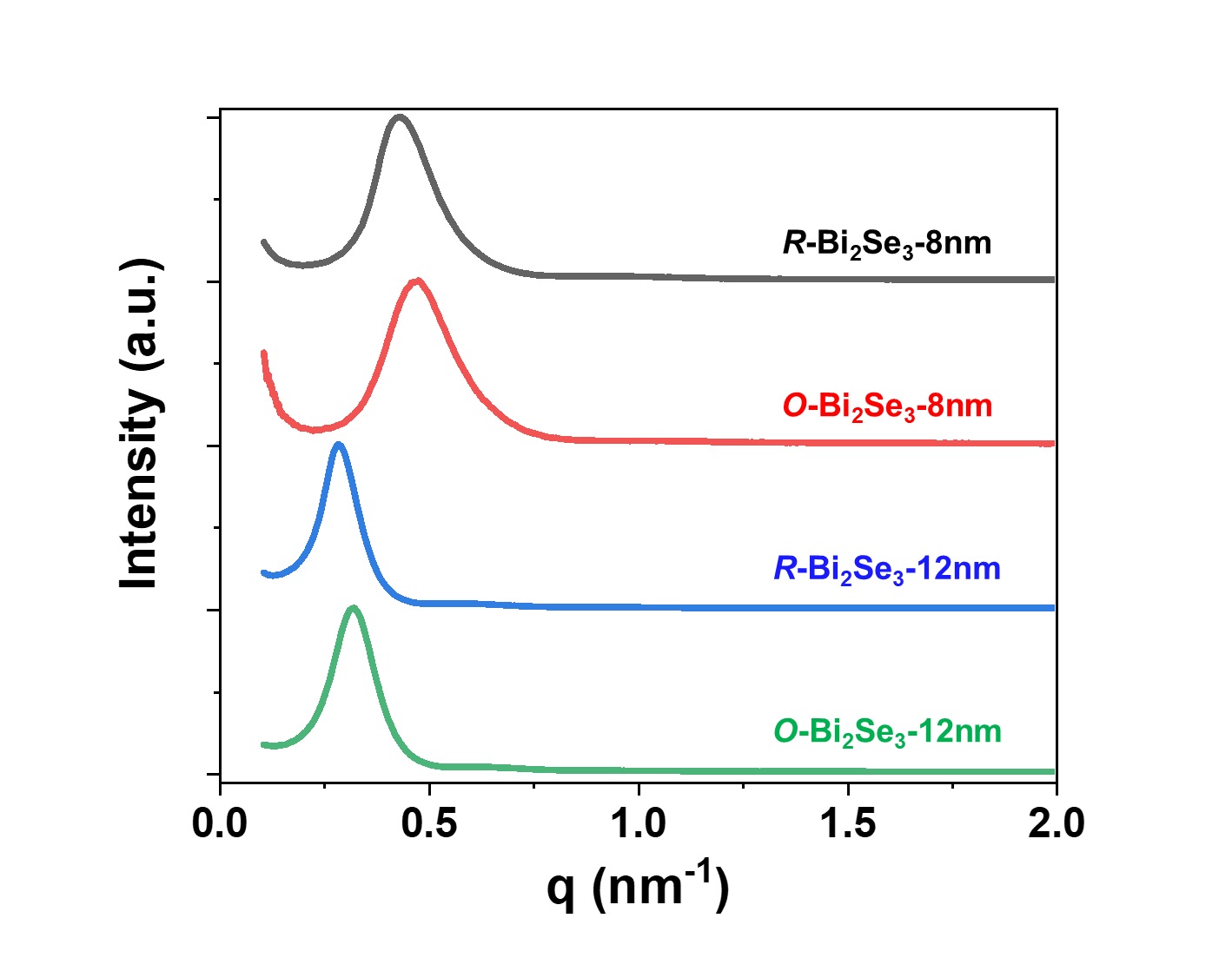


**Figure S8**. SAXS patterns of mesoporous Bi_2_Se_3_ films.


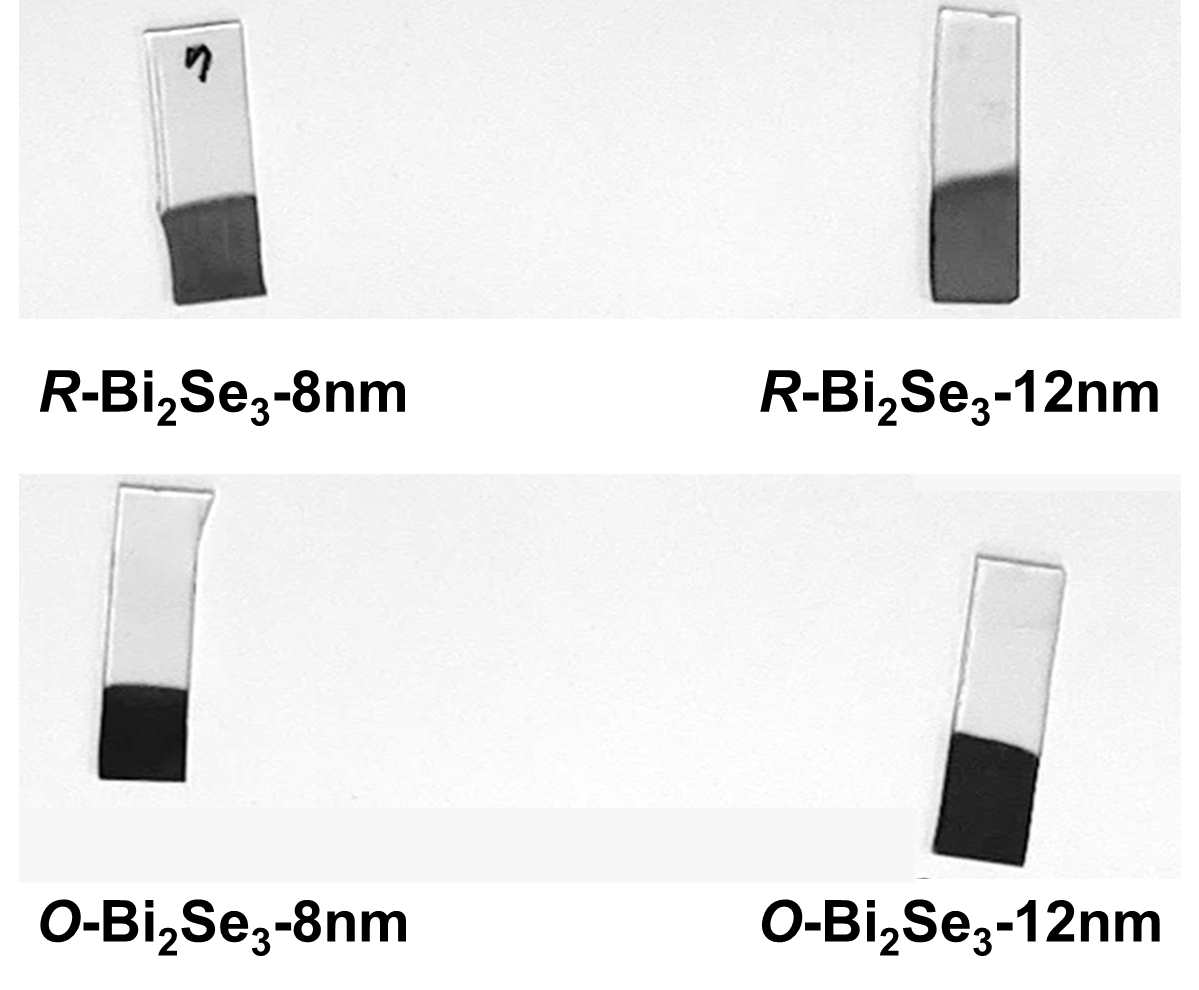


**Figure S9**. Photographs of mesoporous Bi_2_Se_3_ films.


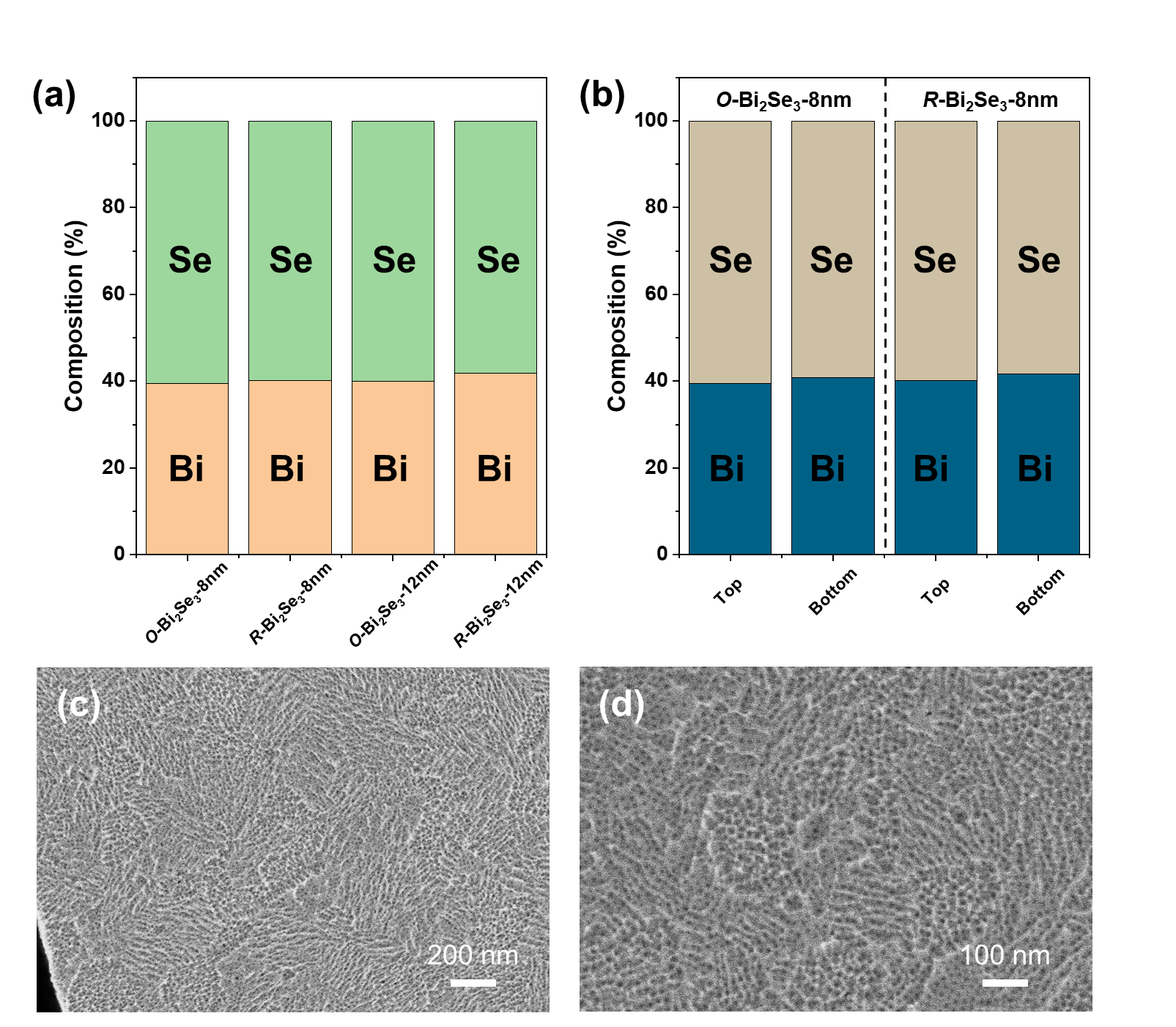


**Figure S10**. (a) Bi and Se composition ratios in Bi_2_Se_3_ films synthesized under different polymer and deposition conditions, as determined by EDX. (b) Bi and Se composition ratios on the top and bottom surfaces of *O*-Bi_2_Se_3_-8nm and *R*-Bi_2_Se_3_-8nm. (c,d) SEM images of the bottom surfaces of (c) *O*-Bi_2_Se_3_-8nm and (d) *R*-Bi_2_Se_3_-8nm.


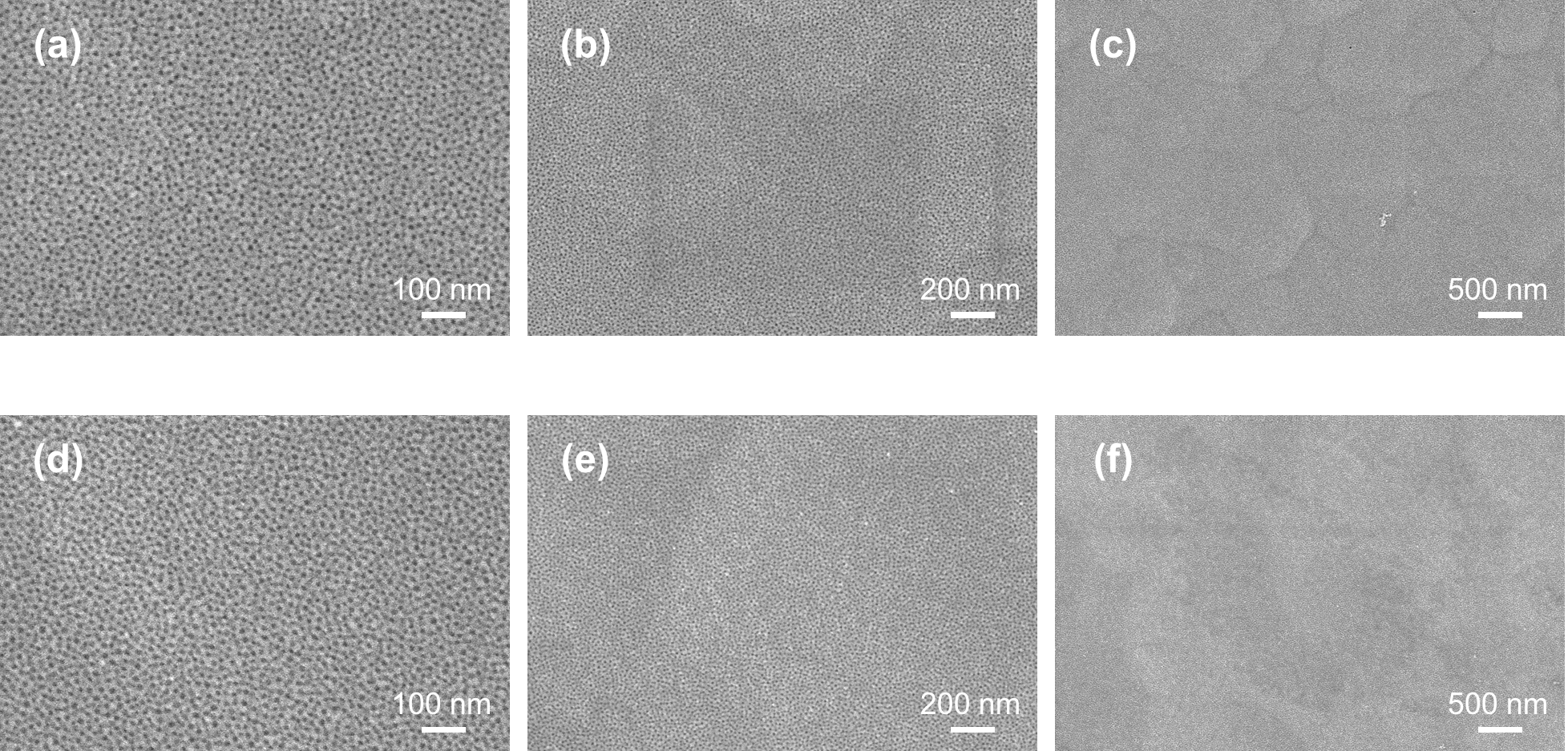


**Figure S11**. SEM images of heat-treated (a-c) *R*-Bi_2_Se_3_-8nm and (d-f) *O*-Bi_2_Se_3_-8nm.


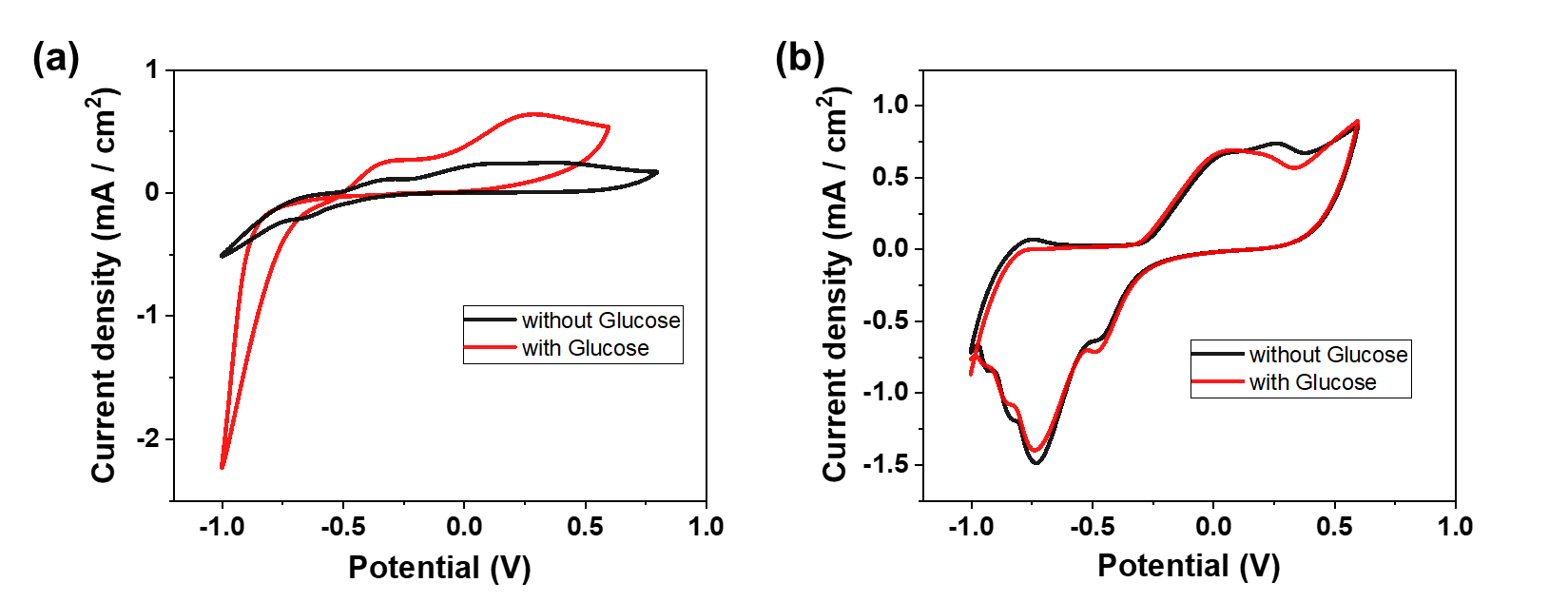


**Figure S12**. CV curves of (a) *R*-Bi_2_Se_3_-8 nm and (b) *O*-Bi_2_Se_3_-8 nm films before and after adding 100 mM glucose solution to 0.1 M phosphate-buffered solution.
